# Central giant cell granuloma of the jaws—long-term clinical and radiological outcomes of surgical and pharmacological management

**DOI:** 10.1007/s00784-024-05585-7

**Published:** 2024-03-07

**Authors:** Tal Capucha, Andrei Krasovsky, Ragda Abdalla-Aslan, Jiriys George Ginini, Dani Noy, Omri Emodi, Adi Rachmiel, Dekel Shilo

**Affiliations:** 1 Oral and Maxillofacial Surgery, Rambam Medical Care Center, Haifa, Israel; 2 Ruth & Bruce Rappaport Faculty of Medicine, Technion-Israel Institute of Technology, Haifa, Israel

**Keywords:** CGCG, Surgery, Steroids, Calcitonin, Jaws, Lesion, Aggressive

## Abstract

**Objectives:**

To compare long-term results of different treatment modalities in central giant cell granuloma of the maxillofacial-skeleton. Primary resection may result in major defects. Alternative treatments include pharmacological agents. As yet there has been no consensus on the use of the variety of treatment options, and few studies have reported clarifying long-term results.

**Materials and methods:**

This retrospective study on 22 patients with 25 lesions evaluated clinical, radiological and histological features, treatment preformed and lesion recurrence. Success was defined as regression/calcification and failure as recurrence, progression or un-responsiveness.

**Results:**

Of the presenting patients, 77% were under age 40. Lesion prevalence was higher in the anterior mandible and left posterior maxilla. Most cases exhibited pain, tooth-mobility or mucosal-expansion. The appearance was predominantly unilocular in the maxilla and multilocular in the mandible, which also exhibited higher prevalence of cortical perforation. Up to 80% of lesions were classified as aggressive.

Intralesional steroids/calcitonin were used in 7 cases. Mean follow-up was 39.8 months. Two cases showed recurrence. In 71% of the cases treated pharmacologically, calcification/regression were observed.

**Conclusions:**

Our analysis indicates better outcomes using a combined approach, including both pharmacological and surgical treatments in large aggressive lesions. Pharmacological treatment resulted in decreased size or well-defined lesions, thus reducing the need for extensive bone resection. Dual treatment with corticosteroids and calcitonin showed no superior outcomes, but a larger cohort should be assessed.

**Clinical Relevance:**

There are several protocols for treatment of central-giant-cell-granuloma lesions, but most are not fully established. It is important to report results that contribute to the establishment of proven protocols. This report attempts to establish the relevance of the combined approach: pharmacological treatment followed by surgical resection.

## Introduction

Central giant cell granuloma (CGCG) is a benign, aggressive, destructive, osteolytic lesion of osteoclastic origin [1]. It is an intraosseous non-neoplastic lesion, consisting of cellular fibrous tissue containing multiple foci of haemorrhage, aggregations of multinucleated giant cells and trabeculae of woven bone [2]. CGCG most commonly occurs in patients under the age of 30, predominantly in females, and it accounts for 7% of all benign tumors of the jaws [1, 2]. The etiology of CGCG is controversial. Jaffe, who first identified the entity, related it to reparative mechanisms following trauma (Jaffe). Another theory associated it with inflammatory responses [3]. Clinically, the lesion is often a painless, slow growing lesion causing expansion of the cortical bone [4]. The lesions are most commonly identified during routine radiologic imaging. Size is highly variable, and resorption or displacement of tooth roots is a common finding. Radiologic appearance may exhibit a unilocular or a multilocular radiolucency [4, 5].

The accepted classification of CGCG is of debate. It consists of an aggressive form and non-aggressive form. Diagnosis is mostly based on clinical and radiological findings. Non-aggressive lesions are painless and slow-growing, whereas aggressive lesions are larger than 5cm, rapid-growing, cause bone expansion, tooth displacement/root resorption, and higher recurrence rates [1, 4, 6, 7]. Aggressive lesions are mostly treated using wider resections whereas more conservative surgical approaches can be used in non-aggressive forms [7–9]. Reconstruction options for the aggressive type may include free bone grafts or vascularized flaps in major resections [8, 9].

Alternative, non-surgical or pre-surgical treatments are becoming more popular in the past two decades. These included calcitonin [7, 10–14], corticosteroids [7, 15, 16], interferon α-2a [7, 17, 18] and, most recently, denosumab [19]. Intralesional corticosteroids were introduced in 1988 and were thought to reduce bone resorption by effecting lysosomal proteases [20]. Later, the use of calcitonin was described [21] and thought to influence lesion progression by inhibiting osteoclast activity. Interferon α-2a was used initially by Kaban et al., in 1999 [18]. The mechanism of action proposed to effect CGCG progression was based on anti-angiogenic properties [22].

These treatments are used to reduce the size of the lesions and in some cases may eradicate them, thus avoiding large resections that result in major functional and aesthetic deformities.

Although various treatment options have been available for some time now, there is no consensus regarding the management of CGCG, and only a few studies have reported consistent long-term results.

The aim of this study was to review and compare long-term results from a cohort of patients with CGCG treated in our institute, surgically and/or pharmacologically.

## Materials and methods

This retrospective study of patients diagnosed with CGCG and treated in our institute between 2000 and 2018 examined demographic details, clinical and radiological features of the lesion and treatment preformed. We documented age, gender, location of the lesion, treatment preformed, clinical and radiological features prior to and following the treatment, and postoperative follow-up including associated morbidity and recurrences. Radiologic features were obtained using computed tomography or panoramic imaging performed prior to and following treatment. These included size, locularity, borders of the lesion, cortical changes/perforation and root displacement/resorption prior to treatment. Clinical signs such as mucosal expansion and pain/paresthesia were also noted. Signs of recurrence as a negative response or bone healing as a positive response were noted. Classification of the lesions as well as the treatment performed, reconstruction, response, morbidity and follow-up duration were recorded. Lesions were classified as aggressive, minor aggressive or non-aggressive according to Chuong et al. and Kaban et al. [6, 23]. Major criteria were size (more than 5 cm) and recurrence. Minor criteria included root resorption, tooth displacement, cortical bone thinning, cortical bone perforation, rapid growth and pain/paraesthesia.

Patients diagnosed with CGCG who were admitted to our hospital and treated in our institute between 2000 and 2018 were evaluated for inclusion in the study. Patients lacking proper documentation, radiographic images or adequate follow-ups were excluded.

Treatment success was defined as bone healing, lesion regression or calcification as response to pharmacological/surgical treatment. Treatment failure was defined as recurrence, no response or progression of the lesion following pharmacological/surgical treatment.

## Results

**Fig. 1:**
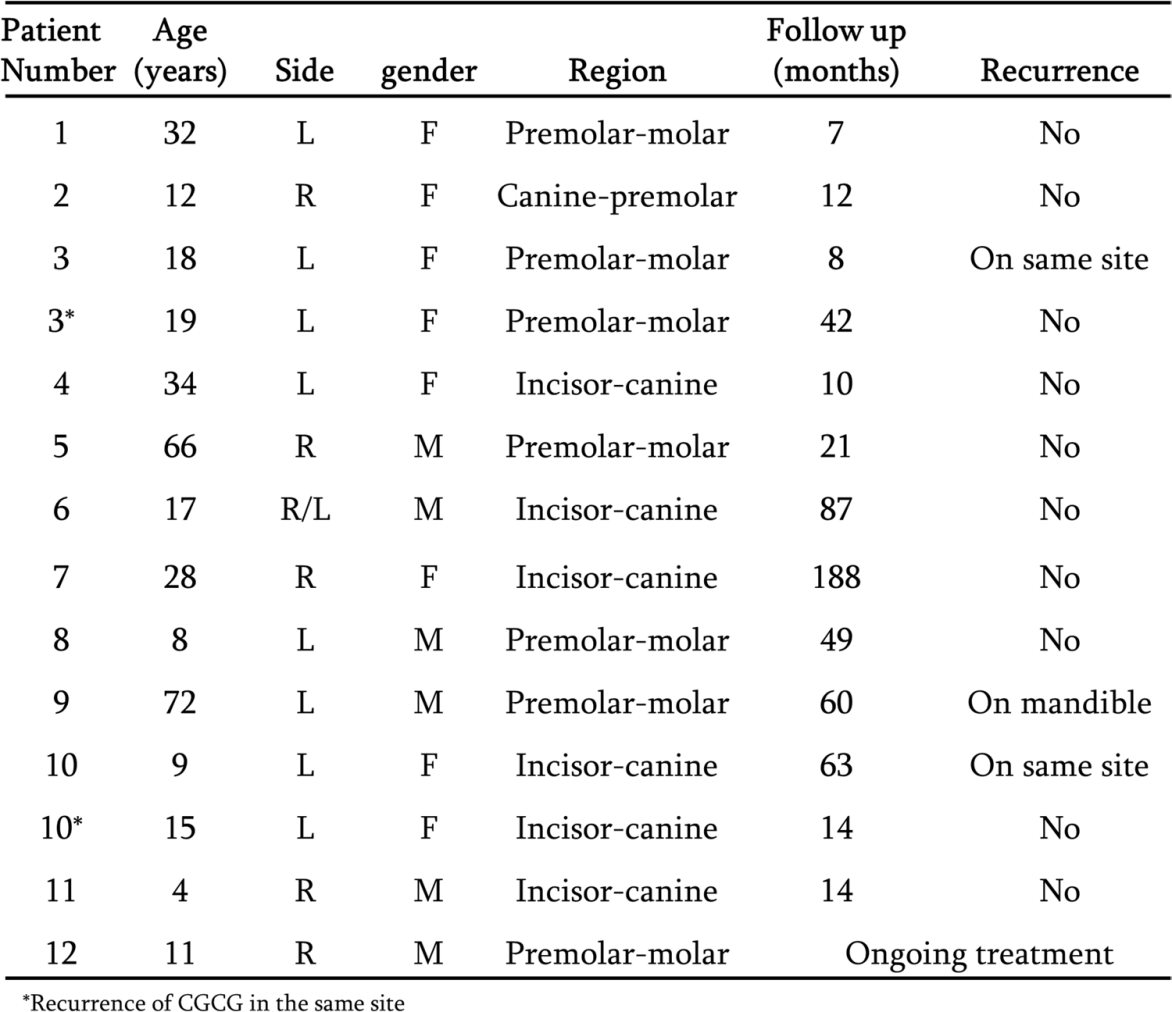
Details of maxillary CGCG lesions. Details include age, location, gender, follow-up and recurrence

**Fig. 2:**
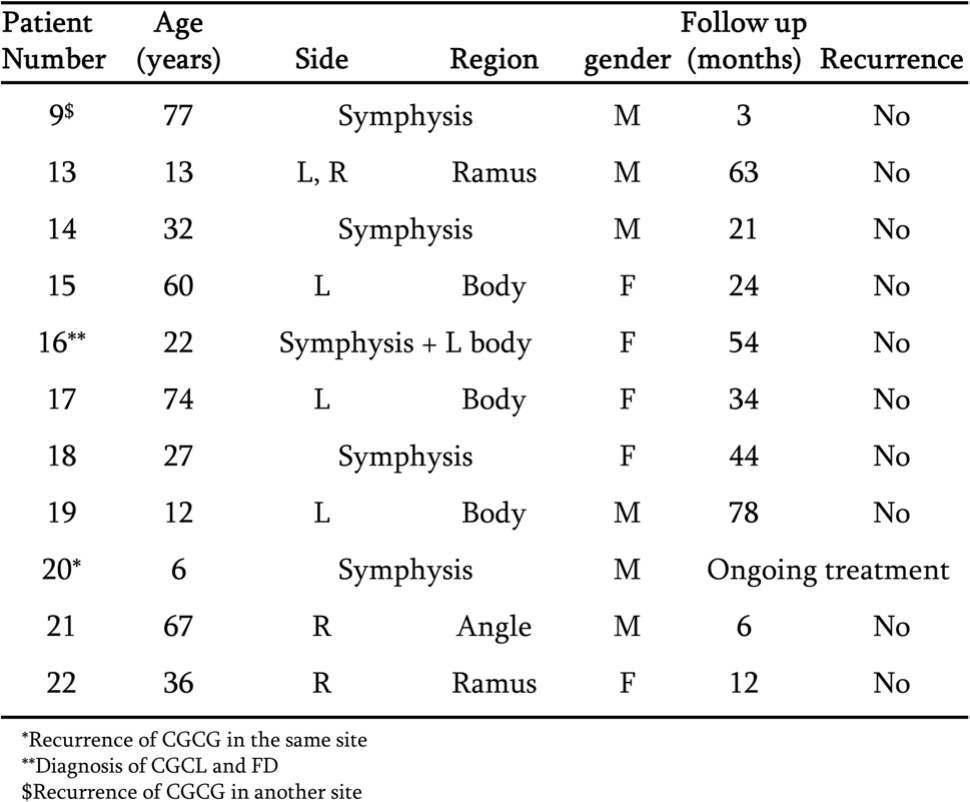
Details of mandibular CGCG lesions. Details include age, location, gender, follow-up and recurrence

Twenty-two patients met the inclusion criteria. Eleven females and eleven males. The mean age was 30-years old, ranging from 4 to 77. Eleven lesions were located in the mandible and fourteen in the maxilla, these included recurrences or a second primary. Details of all the maxillary and mandibular lesions can be observed in Figs. 1 and 2 accordingly and include; age, gender, location, follow-up duration and recurrences. Mean follow-up of the maxillary cases was 46 months. Mean follow-up of the mandibular cases was 31 months. Two recurrences were observed in the maxilla, both in patients aged less than 18, and one second primary was found in the mandible five years following treatment of the maxillary lesion. One recurrence was observed in the mandible in a patient treated at a different hospital by enucleation two years post treatment. He was 6-years old when he presented with the recurrence and is currently being treated pharmacologically with denosumab. Mean age of the maxillary cases was 26. Mean age of the mandibular cases was 39.

**Fig. 3:**
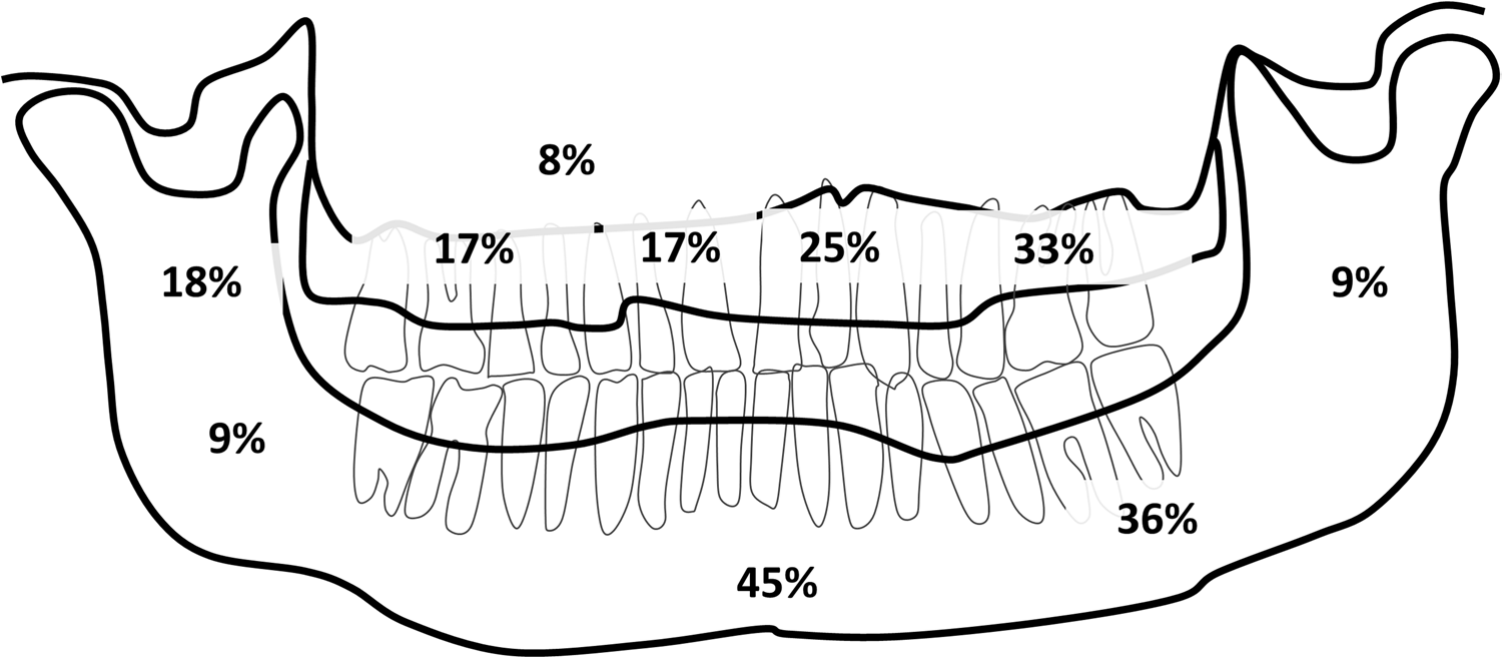
Distribution of Central giant cell lesions in the maxillomandibular complex

Distribution of the lesions in the jaws can be observed in Fig. 3. In the mandible most were observed in the anterior and left body of the mandible. In the maxilla most were located on the anterior and left part of the maxilla.

**Fig. 4:**
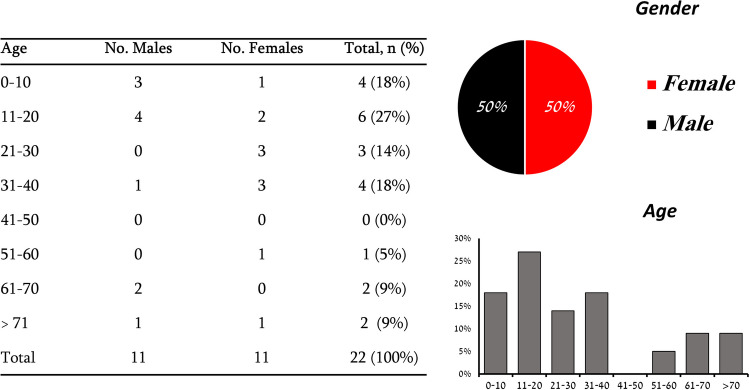
Distribution of Central giant cell lesions according to age and gender

Gender distribution was equal between all cases. Age distribution showed the highest number of cases between 11–20 years old, followed by 0–10 and 31–40 years old (Fig. 4).

**Fig. 5:**
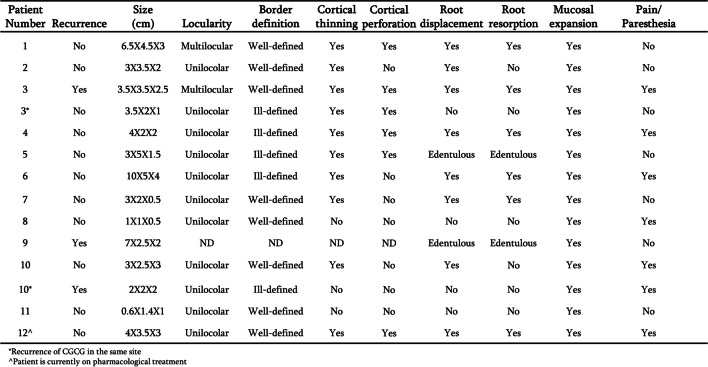
Radiological and clinical characterization of CGCG in the maxilla. Parameters documented included recurrence, size, locularity, border definition, cortical thinning, cortical perforation, root displacement, root resorption, mucosal expansion and pain/paresthesia

**Fig. 6:**
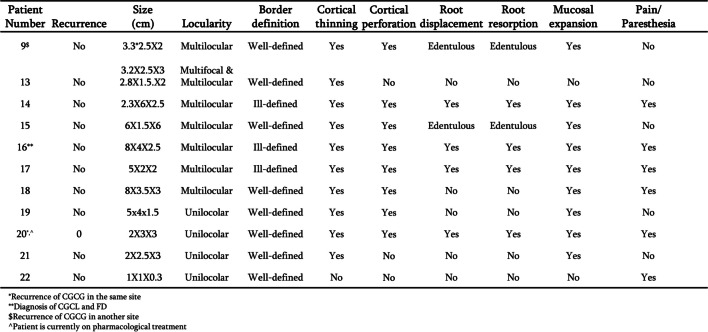
Radiological and clinical characterization of CGCG in the mandible. Parameters documented included recurrence, size, locularity, border definition, cortical thinning, cortical perforation, root displacement, root resorption, mucosal expansion and pain/paresthesia

**Fig. 7:**
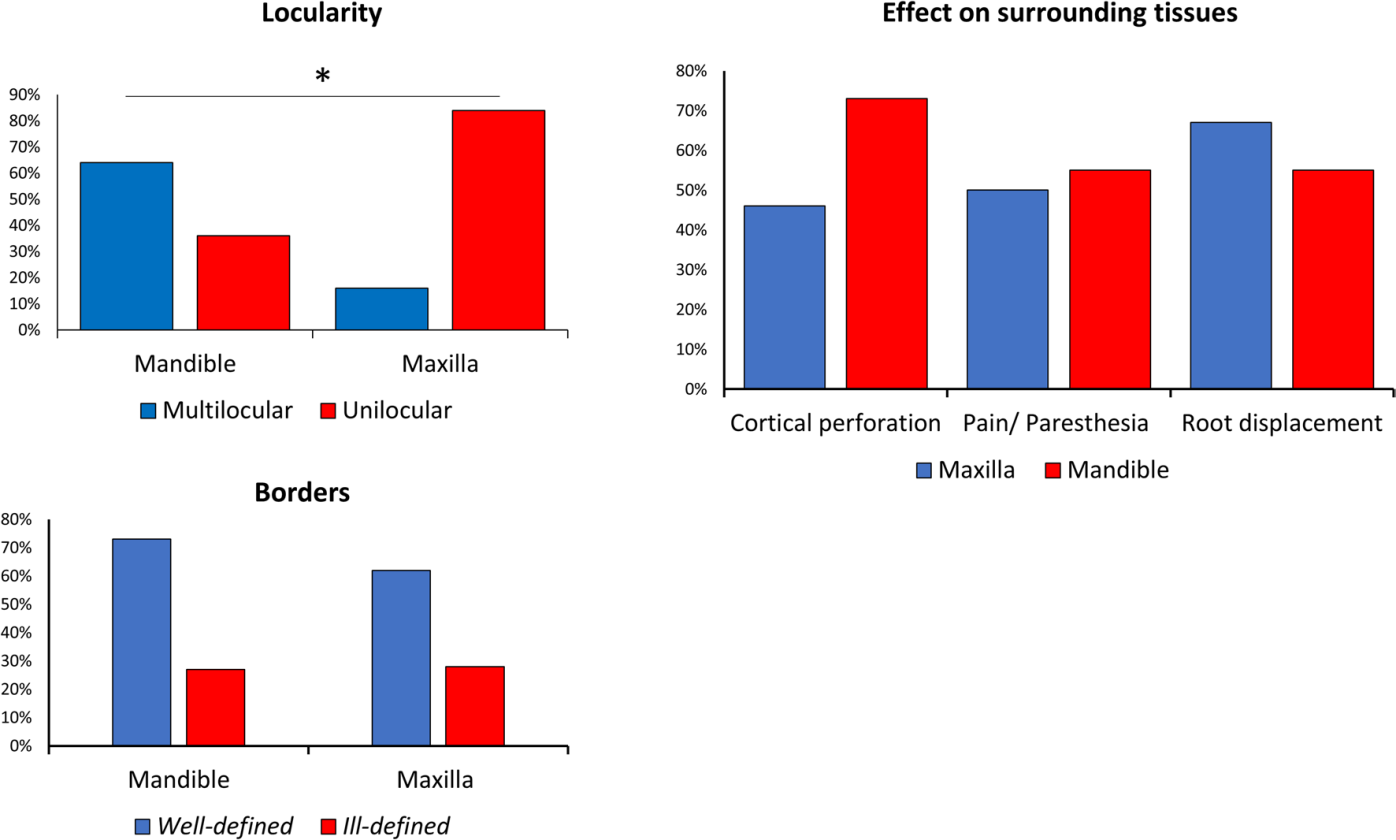
Characteristics of the lesions and their effect on surrounding tissues. Locularity, borders, cortical perforation, pain/paresthesia and root displacement as distributed in the different jaws

Figures 5 and 6 detail the clinical and radiological findings of the lesions in the maxilla and mandible respectively. Figure 7 summarizes these findings. In the mandible most lesions were multilocular, whereas those in the maxilla were mostly unilocular. It is evident that almost all lesions affected the cortex, and perforation occurred at higher rates in the mandible. In both jaws, lesions were more likely to be well-defined.

**Fig. 8:**
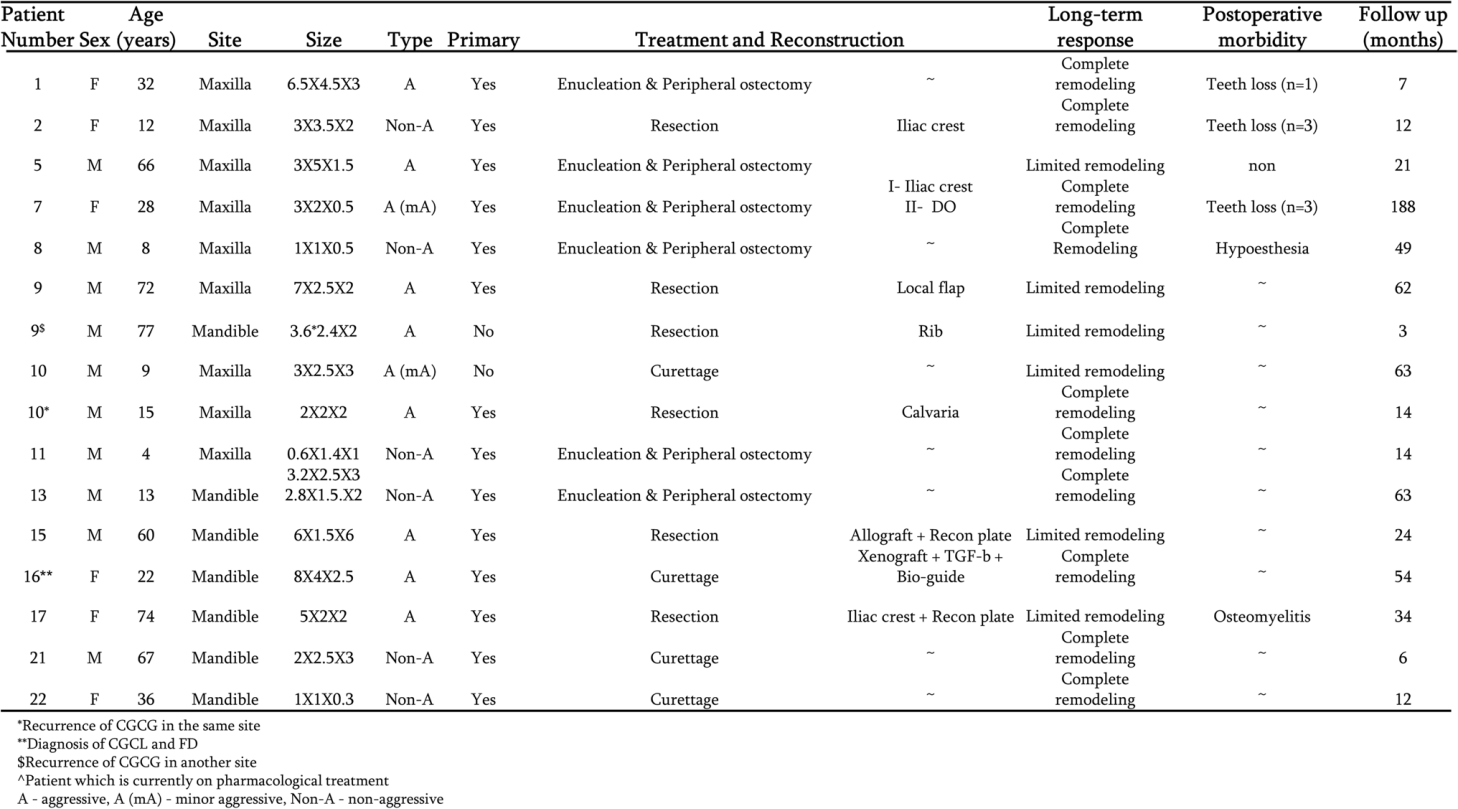
Details regarding the patients treated by surgery alone: location of the lesion, treatment performed, reconstruction if performed, response of the bone to the treatment, morbidity and duration of follow-up

**Fig. 9:**
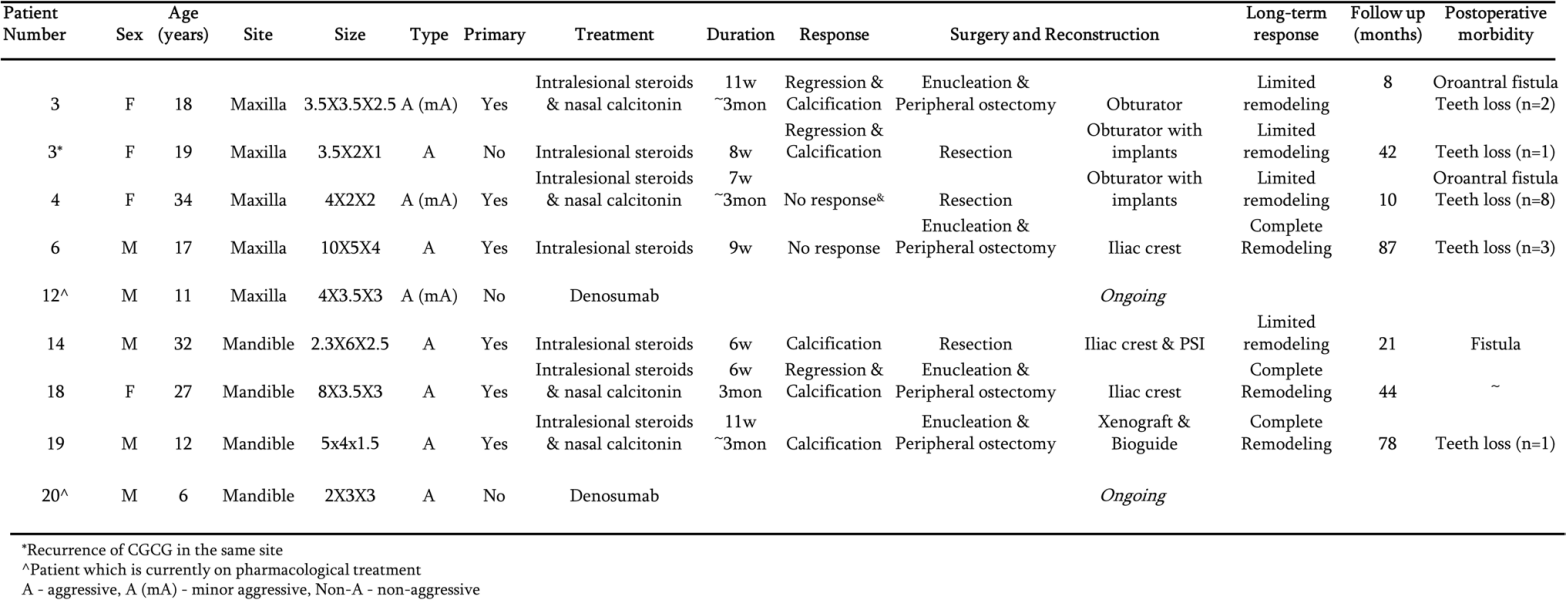
Details regarding the patients treated by pharmacological therapy followed by surgery: location of the lesion, treatment performed, reconstruction if performed, response of the bone to the treatment, morbidity and duration of follow-up

Figures 8 and 9 detail the classification of the lesions, treatment performed, reconstruction performed, response to the treatment, morbidity and follow-up duration in cases treated by surgical means alone or pharmacologically prior to surgery.

**Fig. 10:**
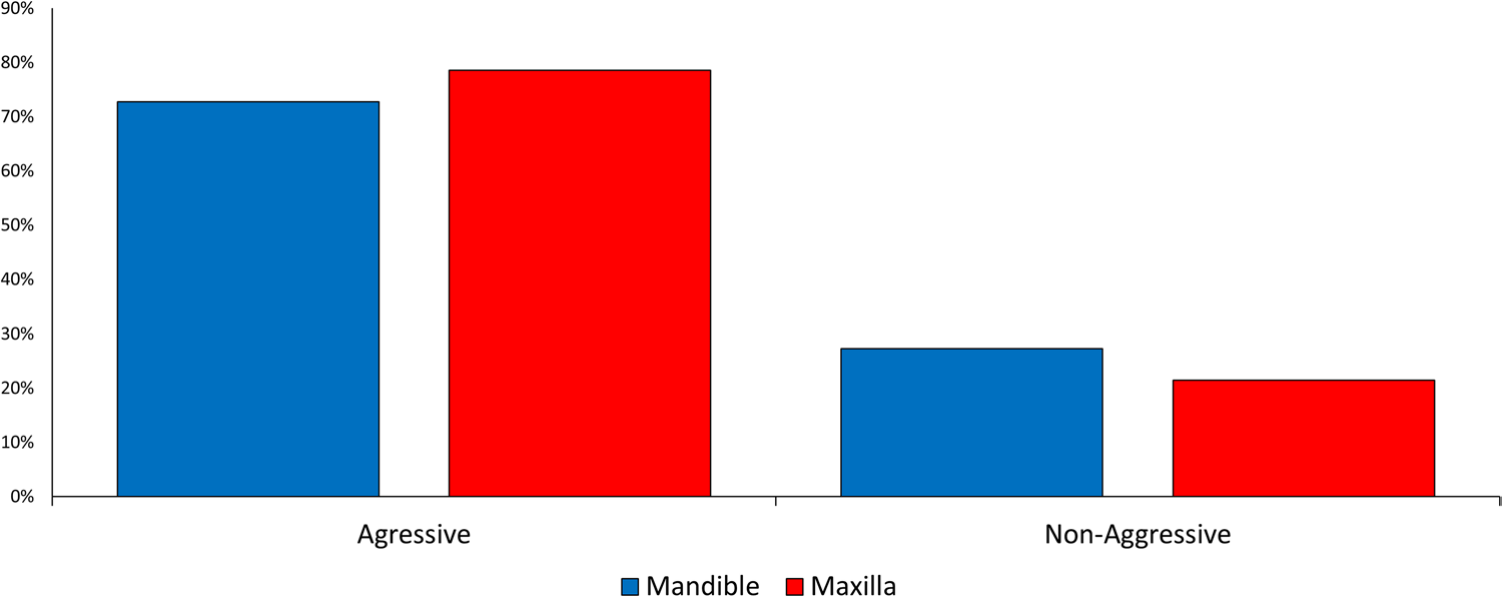
Distribution of aggressive and non-aggressive lesions in the lower and upper jaws

Most lesions were of the aggressive type, both in the mandible and the maxilla (Fig. 10).

For cases treated initially by pharmacological means, 71% exhibited calcification or regression of the lesion, one case did not respond, and one showed progression. Intralesional steroid treatment with or without nasal calcitonin showed good results in shrinking the lesions prior to the surgical treatment.

**Fig. 11:**
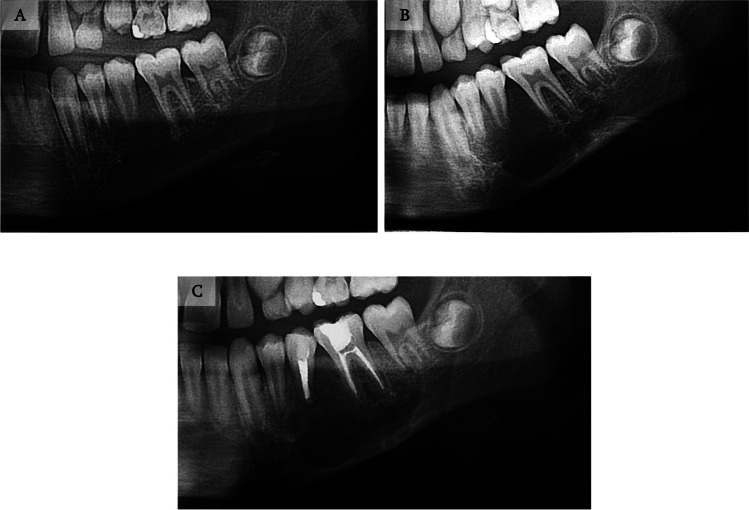
Treatment and follow-up of patient 19. **A** Panoramic radiograph demonstrating a 1.3X2.4 cm lesion located in the mandible of a 12-year-old male. **B** Panoramic radiograph 3-months following diagnosis: lesion enlargement is observed due to delay in treatment. **C** Following pharmacological treatment using intralesional steroids for 11 weeks and nasal calcitonin spray daily.

**Fig. 12:**
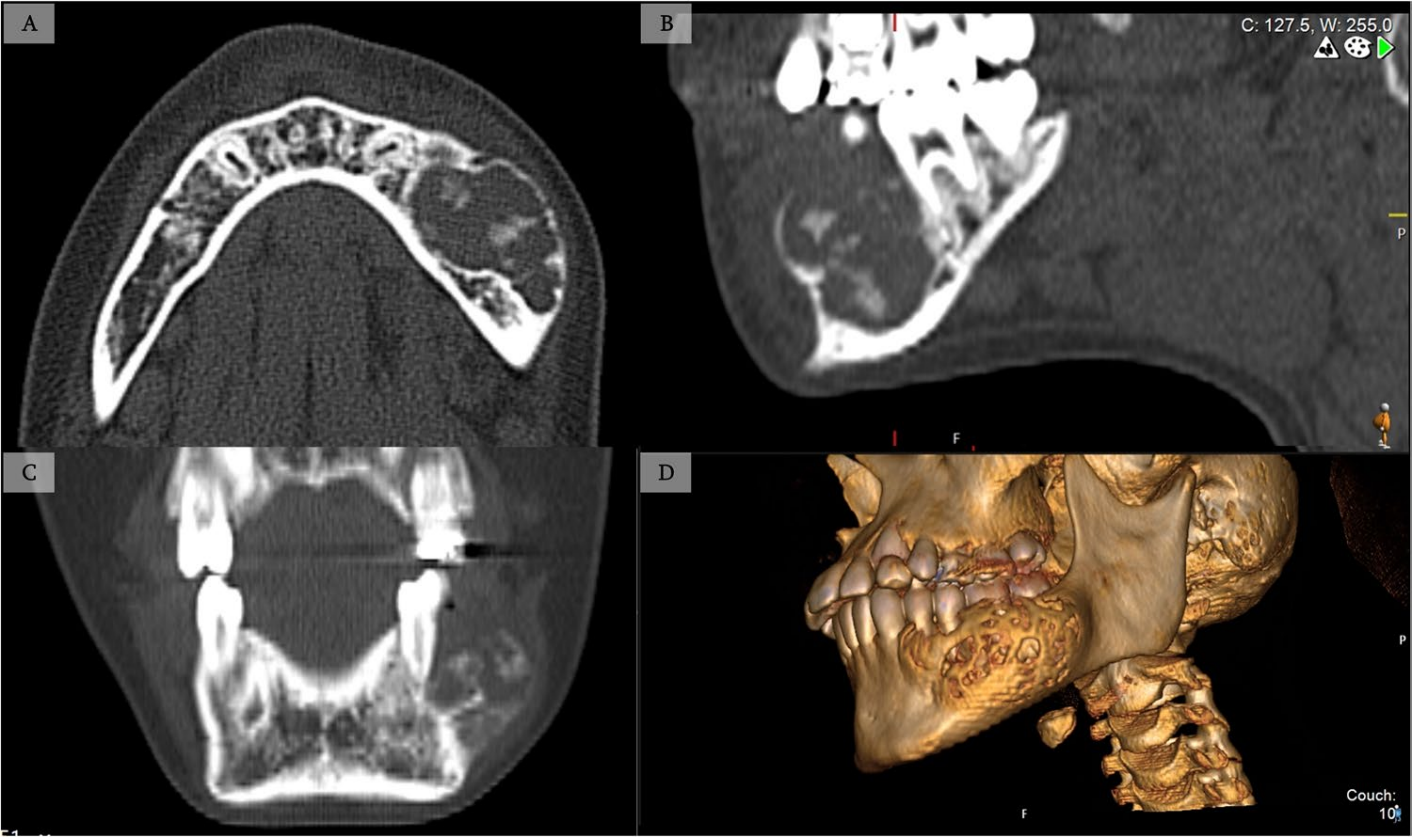
CT imaging following the treatment course of the patient described in Fig. 11. (**A**) Axial (**B**) Sagittal (**C**) Coronal and (**D**) 3D reconstruction, demonstrating the aggressive lesion post pharmacological therapy with intralesional injection and nasal calcitonin as detailed previously

**Fig. 13:**
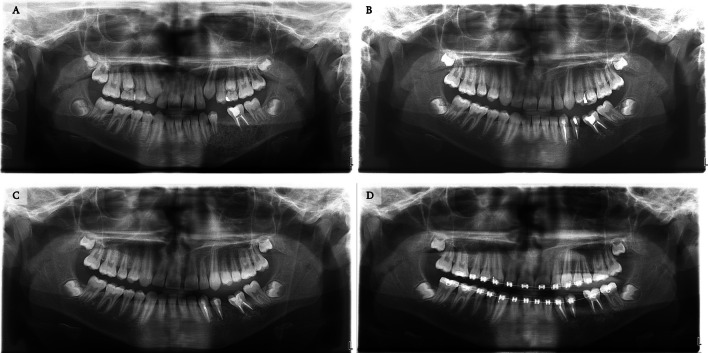
Panoramic radiographs of the treatment and follow-up course of the patient described in Fig. 11. **A** Immediate post-surgical treatment comprised of a partial ostectomy and excision of the aggressive lesion, follow by augmentation with xenograft and a bio-guide membrane. **B**-**D** 1-year, 2-year, and 3-year follow-ups

Figures 11, 12 and 13 document the treatment of patient number 19. The pre-operative radiographs show severe progression of the lesion. Calcification of the lesion is observed following pharmacological treatment. The borders of the lesion can be seen in the CT imaging. This enabled a more conservative surgical approach. Finally, a three-year follow-up indicated no sign of recurrence.

## Discussion

CGCG is a benign, often aggressive lesion. Its progression leads to destruction of the original anatomy and loss of bony tissue, as well as resorption and displacement of dental roots. These lesions are most commonly identified through routine radiographs and thus tend to be large and in need of extensive surgical treatment. Because these lesions are most common in patients under the age of 30, and many times are found in children during their growth period and prior to dento-alvelar maturation, the resulting surgical treatment is associated with high morbidity.

These features and the resulting morbidity led to a search that identified intralesional corticosteroids, calcitonin and Interferon α-2a as pharmacological treatment options for CGCG. Steroids and calcitonin showed some promise, and although not always successful, most patients benefited from the treatment [24, 25]. Interferon, on the other hand, although helpful in some cases, led to major side effects that frequently became intolerable by the patients [26].

The literature does not describe numerous cases that were treated pharmacologically: by 2018, 80 or fewer reliable cases had been treated with calcitonin and a similar number with corticosteroids [27].

The distribution of our cases was almost equal in the mandible and maxilla in contrast to the literature reporting 70% in the mandible. Our sample did not show the predominant occurrence in females observed in the literature. Consistent with the literature, the most prevalent age group in our study was 10–20. Unilocular maxillary lesions were seen more frequently in our study than the literature (84% compared to 62%), as were multilocular mandibular lesions (64% compared to 38%). All minor criteria for the aggressive type were more prevalent in our cohort than reported in the literature: cortical perforation with high incidence in the mandible (73%), pain/paresthesia and root displacement. This explains why the aggressive subtype was dominant in our sample (70–80% of the lesions), both in the mandible and the maxilla. This may be because our institute serves a large rural population in which many patients do not have routine check-ups and thus when diagnosed their lesions are larger.

Some studies in the literature reported that treatment with steroids or calcitonin resulted in complete resolution [24, 28]. Schreuder et al., used calcitonin with or without Interferon α-2a and showed ~ 50% of the lesions did not require further surgical treatment on a large cohort of 33 cases [29]. Most studies reporting the use of pharmacological treatment show improvement in up to 80% of cases, this is in accordance to our results [27]. In our cohort, all cases had to be treated surgically, but 71% responded to the pharmacological treatment, which resulted in smaller lesions that enabled a more conservative surgical approach.

The cases that did not respond to the pharmacological treatment included a patient treated with intralesional steroids and one treated with both intralesional steroids and calcitonin. Although dual therapy does not appear advantageous, the sample size is not large enough for definitive conclusions regarding the difference between treatment modalities.

It is important to mention that the use of monoclonal antibodies for the treatment of CGCG is becoming more prevalent. Following several indications for the efficiency of denosumab as a treatment modality for CGCG more centers began using it as a single [30] or combined [31] method of treatment.

This method was also tested in our institute with strong positive results. Yet they are not free of complications, and serious conditions such as hypercalcemia have been observed [32]. Recent studies suggest higher recurrence rates following cessation of treatment, possibly due to latency of neoplastic cell population or partial curettage secondary to the ossified bone [33].

We do believe these results are sufficient to recommend using pharmacological treatments prior to surgery, but it is important to monitor the response and, if progression is observed, surgical intervention should be performed without any further delay. Determining whether dual therapy is preferable to monotherapy awaits additional evidence.
