# Correction to: Central giant cell granuloma of the jaws—long‑term clinical and radiological outcomes of surgical and pharmacological management

**DOI:** 10.1007/s00784-024-05622-5

**Published:** 2024-04-01

**Authors:** Tal Capucha, Andrei Krasovsky, Ragda Abdalla-Aslan, Jiriys George Ginini, Dani Noy, Omri Emodi, Adi Rachmiel, Dekel Shilo

**Affiliations:** 1 Oral and Maxillofacial Surgery, Rambam Medical Care Center, Haifa, Israel; 2 Ruth & Bruce Rappaport Faculty of Medicine, Technion-Israel Institute of Technology, Haifa, Israel

**Correction to: Annals of Hematology**

10.1007/s00784-024-05585-7

The published paper listed Aslan as the surname and Ragda Abdalla as the first name. The correct first name is Ragda, and the correct surname is Abdalla-Aslan. In addition, the author Dany Noy was misspelled and should be written as Dani Noy. He is also be affiliated with affiliation 2.

The original article has been corrected.

